# Radioligand Therapy with [^177^Lu]Lu-DOTA-TATE or [^177^Lu]Lu-DOTA-TATE and [^90^Y]Y-DOTA-TATE in Patients with Neuroendocrine Neoplasms of Unknown Locations, or Locations Other Than the Midgut and Pancreas as Primaries in a G1, G2 and G3 Grade

**DOI:** 10.3390/ph16091205

**Published:** 2023-08-24

**Authors:** Adam Daniel Durma, Marek Saracyn, Maciej Kołodziej, Katarzyna Jóźwik-Plebanek, Beata Dmochowska, Adrianna Mróz, Wawrzyniec Żmudzki, Grzegorz Kamiński

**Affiliations:** Department of Endocrinology and Radioisotope Therapy, Military Institute of Medicine—National Research Institute, Szaserów 128, 04-141 Warsaw, Poland

**Keywords:** neuroendocrine neoplasms, NEN, RLT, PRRT, 177-Lutetium, 90-Yttrium, G3, lung, unknown primary location

## Abstract

Background: Neuroendocrine neoplasms (NENs) are a rare group of tumors with a different clinical course, prognosis and location. Radioligand therapy (RLT) can be used as a first or second line of treatment. It is registered in gastroenteropancreatic NENs (GEP-NENs) as grades G1 and G2. Tumors with an unknown point of origin, diagnosed outside the gastrointestinal tract and pancreas (non-GEP) or at the G3 grade, remain in the “grey area” of treatment. Materials and Methods: Analysis of 51 patients with NENs who underwent RLT in a single highest reference center from 2018 to 2023 was performed. Treatment was administrated to the patients with neoplasms of unknown origin, non-GEP-NENs, and ones with G3 grade. In total, 35 patients received 177-Lutetium (7.4 GBq), while 16 received 177-Lutetium and 90-Yttrium with equal activities (1.85 + 1.85 GBq). Results: The progression-free survival (PFS) before RLT qualification was 34.39 ± 35.88 months for the whole study group. In subgroups of patients with an unknown tumor location (*n* = 25), the median PFS was 19 months (IQR = 23), with “other” locations (*n* = 21) at 31 months (IQR = 28), and with NEN G3 (*n* = 7) at 18 months (IQR = 40). After RLT, disease stabilization or regression was observed in 42 (87.5% of) patients. RLT did not cause statistical changes in creatinine or GFR values. Hematological parameters (RBC, WBC, PLT, HGB) as well as chromogranin A concentration decreased significantly. There were no statistical differences between both subgroups regarding the type of radioisotope (177-Lutetium vs. 177-Lutetium and 90-Yttrium). After RLT in long-term observation, the median observation time (OT) was 14 months (IQR = 18 months). In patients with progression (*n* = 8), the median PFS was 20 months (IQR = 16 months), while in patients with confirmed death (*n* = 9), the median overall survival (OS) was 8 months (IQR = 14 months). Conclusions: Our study showed that 87.5% of NEN patients with unknown origin, non-GEP-NENs, and those with GEP-NEN G3 grade had benefited from the radioligand therapy. There were no significantly negative impacts on renal parameters. The decrease of bone marrow parameters was acceptable in relation to beneficial disease course. The decrease of chromogranin concentration was confirmed as a predictive factor for disease stabilization or regression.

## 1. Introduction

Neuroendocrine neoplasms (NENs) are a rare group of tumors with an estimated incidence of 350 per million. Their embryological precursor cells are derived from endo- and neuroectodermal tissues [1,2,3]. Despite shared embryological origin, neoplasms do not have identical secretory function, local growth rate, ability to metastasize, clinical course, or treatment prognosis [4,5]. In the European population, NENs are most commonly located in the small intestine, while in Asian countries, the large intestine location predominates. Tumors of gastrointestinal location (stomach, duodenum, small and large intestine), as well as ones derived from the pancreas, are known as “GEP-NENs”. However, if tumors develop virtually in any other part of the body, then they are called “non-GEP-NENs” [6,7,8,9,10]. In 20% of diagnosed cases, the primary tumor location remains unknown. In such cases, the disease is diagnosed solely based on biopsies of metastatic lesions or imaging studies and clinical features [11,12,13]. It seems that the primary starting points of these tumors are the gastrointestinal tract, and the primary lesion itself is simply elusive in a standard diagnostic procedure. This group is usually identified as non-standard in most studies and analyses. 

The first line of treatment for NEN is surgical, followed by treatment with use of long-acting somatostatin analogues (SSAs) [14]. Currently, two registered pharmaceuticals are available: lanreotide and octreotide. The use of somatostatin analogues (SSAs) is also the most common first-line treatment approach for cases involving inoperable lesions. Treatment can be applied after confirming the presence of somatostatin receptors in functional tests (somatostatin receptor scintigraphy with ^99m^Tc or less often ^111^In, or PET/CT with ^68^Ga). In the subsequent lines of treatment, and when the expression of somatostatin receptors is confirmed, radioligand therapy (RLT) can be applied. RLT, previously known as peptide receptor radionuclide therapy (PRRT), and commonly known as therapy with “hot analogues”, is the preferred therapeutic method in stages G1, G2, or G3, with confirmed somatostatin receptor expression in the aforementioned imaging studies [14]. The worldwide guidelines prefer 177-Lutetium in monotherapy—([^177^Lu]Lu-DOTA-TATE), but the therapeutic option remains tandem therapy with a mixture of 177-Lutetium and 90-Yttrium isotopes ([^177^Lu]Lu-DOTA-TATE and [^90^Y]Y-DOTATATE) [15,16]. The differences between lutetium and yttrium isotopes are presented in Table 1. The higher energy and range of lutetium is considered to be a greater risk factor for possible complications, mainly in the kidneys and bone marrow. However, due to its greater energy and range, the potential beneficial impact on the treatment of large tumors/metastases may be higher [17,18,19].

In 2017, the only radiopharmaceutical for NEN treatment (Lutathera^®^) was registered in Europe by the European Medicines Agency (EMA), and in 2018, in the USA, the Federal Drug Administration (FDA). It is a radiopharmaceutical of [^177^Lu]Lu-DOTA-TATE with an activity of 7.4 GBq used to treat non-operable or metastatic, progressive, well-differentiated (G1 and G2) gastroenteropancreatic neuroendocrine neoplasms (GEP-NENs), with confirmed somatostatin receptor expression in adults [20]. It is administered in four courses (administrations) at 8-week intervals; treatment maintenance is conducted with a long-acting somatostatin analogue (lanreotide 120 mg or octreotide 30 mg) every 4 weeks [20,21]. The other therapeutic options for patients with NEN include chemotherapy, which is preferred for tumors with a higher proliferation index (Ki-67), such as NEN G3 or neuroendocrine carcinomas (NECs), with a recommended two-component scheme such as capecitabine plus temozolomide (CAPTEM) or carboplatin plus etoposide [14,22,23,24,25]. Equivalent therapeutic options are targeted therapies: tyrosine kinase inhibitor (TKI)—sunitinib or a selective inhibitor of m-TOR (mammalian target of rapamycin)—everolimus, However, they are limited by possible complications and side effects [26,27].

## 2. Results

**Figure 1:**
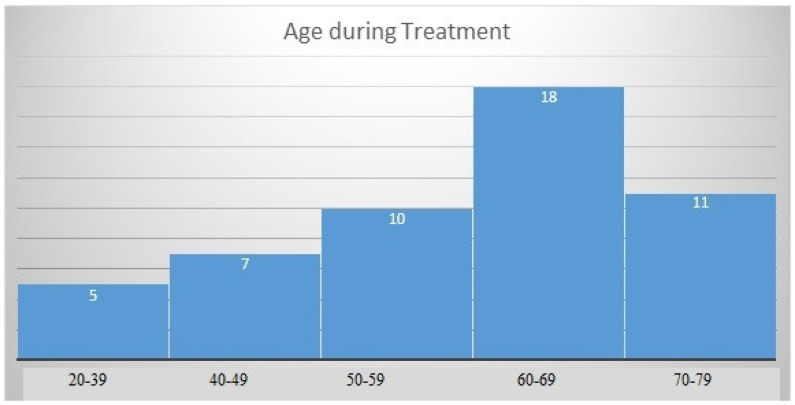
Age distribution of the study group.

**Figure 2:**
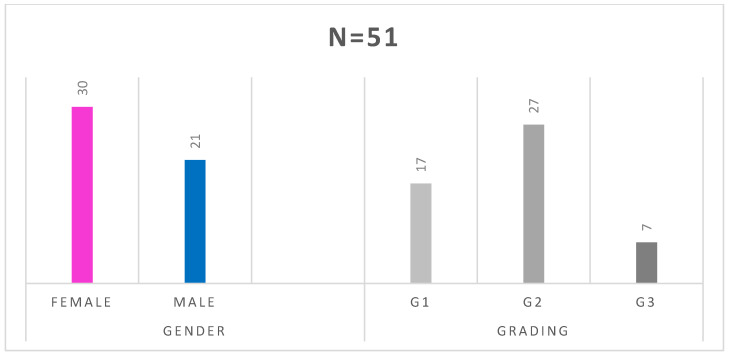
Gender and NEN grading distribution of the study group.

**Figure 3:**
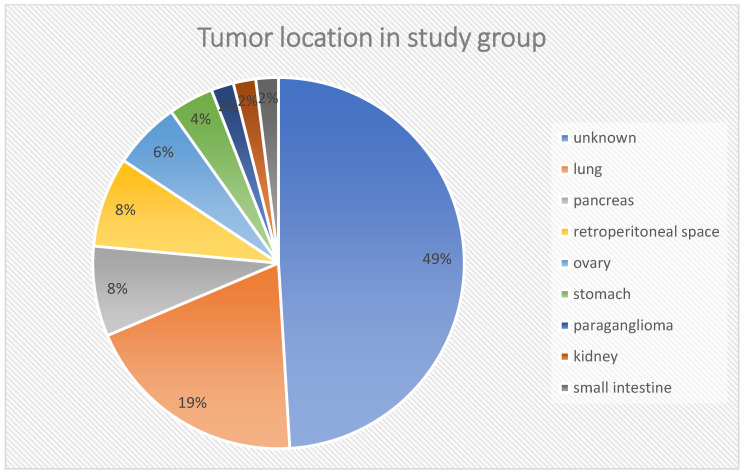
Tumor locations in the study group.

The mean age of patients at diagnosis of neuroendocrine tumors was 58.53 (SD = 12.44) years, with a normal distribution of the group structure (Figure 1). The studied group consisted of 30 women and 21 men. In 17 patients, the tumor was Grade 1(G1), in 27—G2, and in 7—G3 (Figure 2). In the histopathological analysis of the tumors (biopsy/operation), the mean proliferation index (Ki-67) was 9.82 ± 8.69%. The location of the primary tumor was unknown in 25 cases, and in 21 patients, the primary location was outside the gastrointestinal tract and pancreas, i.e., in the lungs (*n* = 10–19.6%), retroperitoneal space (*n* = 4–7.8%), ovaries (*n* = 3–5.9%), stomach (*n* = 2–3.9%), kidney (*n* = 1–2%) or were paragangliomas (*n* = 1–2%). These locations are referred in the discussion as “other” locations. The rest of cases were NEN G3 (pancreas, *n* = 4; small intestine, *n* = 1). The carcinoid syndrome occurred in 23 patients, 1 patient had metastatic paraganglioma, and 1 had a hormone-secreting NEN growth (GH-oma). Twenty-six patients (51%) had hormonally inactive tumors. Details of the groups and subgroups are presented in Table 2 and Table 3, and Figure 3.

**Figure 4:**
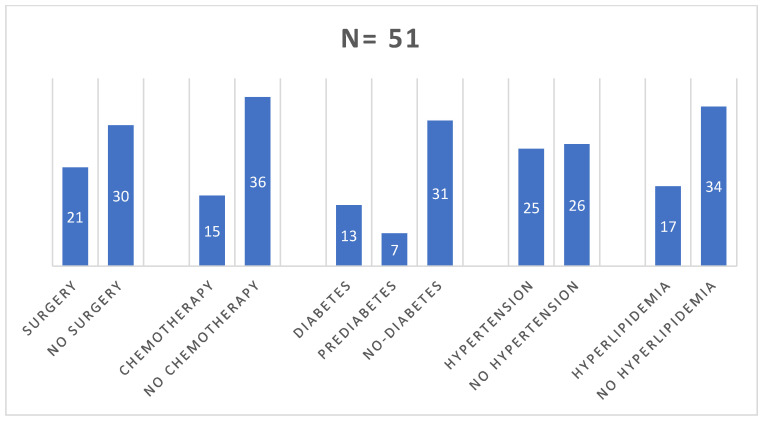
Epidemiological analysis of the study group.

**Figure 5:**
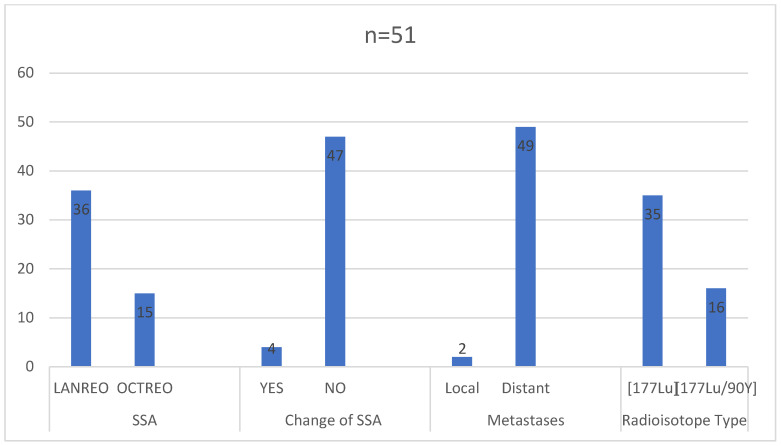
Treatment analysis of the study group.

In the study group, the primary tumor staging or other medical aspects prevented surgical treatment in 30 (58.82%) patients. Chemotherapy was administered to 15 (29.41%) patients prior to initiating radioisotope treatment. Initially, about 40% of patients had pre-diabetes or diabetes, 50% had hypertension, and 33% had hyperlipidemia (Figure 4). The majority of patients received monthly injections of lanreotide (36 received Somatuline Autogel^®^ 120 mg and 15 received Sandostatin LAR^®^/Okteva^®^ 30 mg). Only four patients had a change in the somatostatin analogue used during treatment (three from octreotide to lanreotide, one from lanreotide to octreotide). At the time of qualification, 49 (89.1%) patients were found to have disseminated cancer, while 2 (1.9%) showed increased local and regional progression (Figure 5).

Out of the total, 35 patients (68.63%) underwent treatment involving 177-Lutetium (7.4 GBq). This treatment was administered intravenously (i.v.) in four cycles with intervals of 10 to 12 weeks. On the other hand, 16 patients (31.37%) underwent tandem therapy with a combination of 177-Lutetium and 90-Yttrium in equal activities (1.85 GBq + 1.85 GBq). Similar to the first group, the tandem therapy was delivered through intravenous (i.v.) administration in four cycles with 10 to 12 weeks between each administration.

Progression-free survival (PFS), defined as the time from diagnosis and primary treatment (surgical and/or long-acting analogue) to RLT start, was 34.39 ± 35.88 months for the entire study group (*n* = 51). In subgroups of patients with neuroendocrine neoplasms of unknown origin (*n* = 25), or “other” location (*n* = 21) and Grade G3 tumors (*n* = 7), the median PFS was 19 (interquartile range—IQR = 23), 31 months (IQR = 28), and 18 (IQR = 40), respectively.

In the short-time analysis of treatment results, 48 patients were included. The remaining three were excluded due to incomplete results or receiving only one course of treatment. Patients underwent an average of 3.57 ± 1.25 treatment courses. Initial disease stabilization (directly after RLT) was observed in 33 (68.75%) patients, regression in 9 (18.75%), and only 6 (12.5%) patients had progression during treatment. Details on the subgroup of patients with progression are described in Table 4.

**Figure 6:**
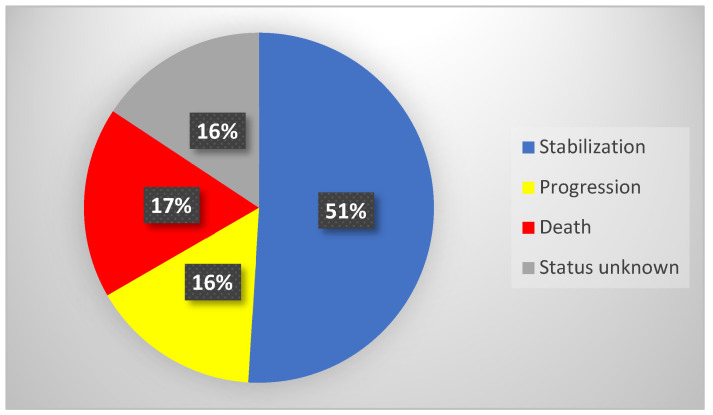
Long-term observation data of the group—percentage of treatment outcome.

Overall, the medium observation time of the study group (*n* = 51) after RLT was 14 months (IQR = 18 months). Stabilization of the neoplastic disease was confirmed in 26 individuals (51%), with the median observation time (OT) in this subgroup being 15 months (IQR = 17 months). Progression was confirmed in eight patients (16%), with the median progression free survival time being 20 months (IQR = 16 months). Death was confirmed in nine patients (17%)—their median time of overall survival (OS) was 8 months (IQR = 14 months). In eight patients, the accurate status of the disease was unknown due to lack of contact (neither with patients nor with their families). Detailed data of treatment outcomes are presented in Table 5 and Figure 6.

As outlined above, analysis of possible short-term complications was performed on a subgroup 48 patients. The group was divided into two subgroups: patients who received 177-Lutetium in monotherapy (*n* = 32) and those who received tandem therapy with 177-Lutetium and 90-Yttrium (*n* = 16). The results were analyzed at two time points: before the first (A) and before the last (B) radioisotope administration (Table 6). The reliable analysis of long-term complication was not possible due to lack of agreement to deliver all required laboratory results. Analysis of the impact of the therapy on renal parameters showed a mean decrease in glomerular filtration rate (GFR) in the group receiving lutetium of 1.22 mL/min/1.73 m^2^ and an increase in tandem therapy of 0.44 mL/min/1.73 m^2^, although the results of renal parameters were not statistically significant. Statistical significance was observed in the decrease of the number of cells of all bone marrow lines, as well as in the hemoglobin concentration, regardless of the administered radioisotope (Table 6). Comparison of patients treated with different types of radioisotopes, despite slightly deeper decreases in erythrocytes counts (−0.39 vs. −0.51 million/µL), leukocytes (−1.51 vs. −2.32 10^3^/µL), and hemoglobin (−0.61 vs. −0.87 g/dL) in the case of tandem therapy, did not show a statistically significant difference (Table 6). A statistically significant decrease in CgA concentration was observed after RLT (Table 6). These values correlated with imaging results, but again, no statistically significant differences were found between both therapies (ΔCgA = −31 ng/mL for 177-Lutetium vs. −49 ng/mL for 177-Lutetium and 90-Yttrium) (Table 7).

## 3. Discussion

Our study aimed to show the effect of RLT in patients with NENs who were not able to qualify for standard and registered radiopharmaceutical treatment. Depending on local law, patients with neuroendocrine neoplasms of unknown primary location, non-midgut and non-pancreatic location, as well as ones with a G3 grade (with preserved somatostatin receptor expression) may be excluded from potentially effective, safe, and available treatment. As radioligand therapy with the use of [^177^Lu]Lu-DOTA-TATE or [^177^Lu]Lu-DOTATATE and [^90^Y]Y-DOTA-TATE carries a lower number of acceptable side-effects, the benefits from the treatment almost always outweighs the possible complications [28,29,30,31,32,33,34]. In our study, immediately after RLT, nearly 9 out of 10 patients benefited from the treatment, showing regression or stabilization of the disease and tumor growth.

The analysis of the medical history of the entire study group (*n* = 51) revealed that mean Progression-Free Survival [PFS] during the treatment with SSAs before RLT was 34.39 months. Among the three analyzed subgroups, the longest time of disease stabilization was observed in tumors located outside the midgut and pancreas. The short-term evaluation of treatment outcomes during the last course of RLT demonstrated that almost ninety percent of patients benefited from the therapy. In long-term observation, more than a year after treatment, over half of the patients showed stabilization of the neoplastic disease, confirming RLT as a reliable and efficient method of NENs treatment, prolonging the patient’s life [35].

In the subgroup of patients who experienced progression during the treatment (*n* = 6), the primary tumor location varied, encompassing practically every possible location. Similarly, the tumor grading and the marker of proliferation (Ki-67 index) were significantly different (G1-G3; Ki-67 range: 2–25%). Patients in this subgroup also had different genders (four males vs. two females), presented symptoms of carcinoid syndrome (four yes vs. two no), and underwent different types of therapy (four received 177-Lutetium; vs. two receiving 177-Lutetium and 90-Yttrium). Due to all types of NENs progress, regardless of their primary location, function, grading, staging, and type of treatment, a hypothesis could be proposed that all patients with NEN progression could potentially benefit from RLT. Therefore, it seems relevant to consider expanding the indications for available radiopharmaceuticals to more treatment options, and the use of RLT should be considered as an “off-label” treatment. 

An interesting fact is that during the assessment of potential complications in the study group, we observed a decrease in renal function parameters in both the 177-Lutetium monotherapy and 177-Lutetium and 90-Yttrium therapy subgroups, however, the results were not statistically significant. This may be due to the short observation period and the initially normal GFR values. In another prospective study conducted by the authors of this paper, the permanent reduction of GFR up to 10% in long-term follow-up was observed [36]. Some other papers confirmed these results, also proving a low grade of potential complications [15,37,38]. Although we did not notice a statistical decrease in renal parameters in the subgroup of patients described in the manuscript, it should be emphasized that the observed decrease in glomerular filtration rate and the increase of creatinine concentration exceeded the natural decline of GFR associated with age. The natural annual decrease of renal filtration in the Central European population (>40 years) is about 1%. Therefore, the impact of radioligand therapy remains non-neutral for kidney function, and this aspect must always be taken into consideration before introducing treatment. In patients with a lower initial GFR, it should be especially closely monitored, adhering to the principle “Primum non nocere”—“First, do no harm”. However, the positive effects of radioligand therapy still outweigh the possible side effects of this treatment [39,40,41].

While decrease of renal parameters was not significant, a decrease of all blood cells count was. RLT affected all bone marrow lines, i.e., erythrocytes (RBC), leukocytes (WBC), thrombocytes (PLT), as well as hemoglobin (HGB) concentration. The results of the study group were similar to ones observed in previous studies. Despite reaching statistical significancy, the changes in described parameters were not clinically significant [42,43]. 

The impact of radioligand therapy on the bone marrow is mainly caused by the circulation of the radioisotope in the blood and the action of emitted radiation (β and γ). The cumulative effect of radiation on bone marrow cells technically depends on the radioisotope time of bloodstream circulation. It might be possible that intensive body hydration and increasing diuresis can be protective factors during the treatment, but there is a lack of high-value data to confirm this possibility [44]. 

A previous large study by Kesavan et al., which assessed 2225 NEN patients, of whom 2104 were treated with RLT alone and 121 with combined RLT and chemotherapy, showed short-term bone marrow damage only in 221 patients (9.93%) within the whole study group. In the subgroup treated with RLT as monotherapy, the complication occurred in 213 (10.12%) individuals, while in the RLT plus chemotherapy subgroup, it occurred in 8 (6.61%) patients. The acute complication reached a maximum of 3 to 4 on the WHO CTCEA scale and mainly manifested as a self-limiting decrease in platelet count. Myelodysplastic syndrome was found only in 31 (1.4%) patients in the whole group. The risk factors in the study were an impaired base renal function, older age (>70 years), and previous chemotherapy. It is also worth noting that the two latter risk factors also influenced GFR values [45]. 

Chromogranin A (CgA) is a non-specific marker of NENs, which is helpful in the diagnosis of the disease and could be used for monitoring of the treatment effectiveness. Nevertheless, because of lack of standardization, common use of this parameter is limited. It remains a clinical problem, because laboratory results performed in different centers can differ depending on the laboratory, method and reagents used, and may not be comparable. Notwithstanding the above, in our study, all patients were tested in one center, using the same ELISA test. An observed decrease of CgA in the study group (as well as in both subgroups: 177-Lutetium vs. 177-Lutetium and 90-Yttrium) correlated with clinical and imaging results, confirming the stabilization or regression of the tumor. We also observed a more marked decrease of mean CgA concentration in the group treated with tandem therapy (compared to monotherapy), but results were non-significant. This phenomenon was previously confirmed in the results of other studies, which pointed out the usefulness of the parameter as an indicator of disease progression and overall survival [46,47,48,49,50].

In our study, we did not observe statistical differences between subgroups receiving 177-Lutetium in monotherapy and tandem therapy with 177-Lutetium and 90-Yttrium. Some slight differences, such as a deeper decrease in the number of blood cells, GFR, or CgA in tandem therapy, corresponded with the known physical characteristic of 90-Yttrium, described thoroughly in the introduction. However, it must be pointed out that no statistical differences were found between both subgroups in terms of any evaluated parameter.

Despite the lack of a control group description (utilizing a placebo or continuing previous treatment), which is a minor limitation of the presented study, we believe that suggesting the creation of such a group in the context of evaluating PFS, OS, or quality of life (QoL) would be on the border of professional ethics. Considering the initially observed progression, RLT seems to be one of the most valuable therapeutic options for those patients.

The global increase of neuroendocrine tumors’ incidence is still not fully explained, but may result from changes in the modern environment, as well as of more sensitive diagnostic methods and the development of knowledge about this rare group of tumors [49,50,51,52]. Based on the epidemiological data, in the near future, the care of patients with NEN will become a significant challenge for clinicians and the healthcare systems in all countries. With access to all therapeutic methods, as well as better knowledge about the disease, it will be possible to individualize therapy and achieve better progression free survival and overall survival ratios. Thus, the studies on NENs, radiopharmaceuticals and their complications should be performed further to improve their treatment.

## 4. Materials and Methods

During a 5-year observation, 51 patients, who did not qualify for treatment with the registered lutetium radiopharmaceutical, were treated with radioligand therapy for neuroendocrine tumors at the Department of Endocrinology and Radioisotope Therapy of the Military Institute of Medicine—National Research Institute (MIM-NRI), Warsaw, Poland. Those patients received locally produced lutetium (LutaPol, POLATOM, Poland; GMP medicinal product authorization No. 22081) in monotherapy with 7.4 GBq activity of [^177^Lu]Lu-DOTA-TATE; or tandem therapy (LutaPol and ItraPol, POLATOM, Poland GMP medicinal product authorization No. 22081 and 22069, respectively), where activities were as follows: 1.85 GBq of [^177^Lu]Lu-DOTA-TATE + 1.85 GBq of [^90^Y]Y-DOTA-TATE. Both radioisotopes were administrated in four IV cycles with 10–12-week intervals. The study was conducted in accordance with the Declaration of Helsinki, and approved by the Ethics Committee of Military Medical Chamber, decision number 154/17. During each hospitalization, intravenous nephroprotection was administered during (1000 mL) and the day after (500 mL) radioisotope administration for patients who had received amino acid infusions (Nephrotec^®^, Fresenius Kabi, Poland). Clinical outcomes of the treatment as well as epidemiological/demographic data and laboratory results were analyzed. 

### 4.1. Laboratory Tests

Blood samples were collected with BD Vacutainer Tests in the Department of Endocrinology and Radioisotope Therapy, and analyzed in the Department of Medical Diagnostics, Military Institute of Medicine—National Research Institute, Warsaw, Poland. Biochemistry was analyzed using the Roche Diagnostics Assays (Germany) and Hitachi High-Tech Corporation COBAS c503 PRO Automatic Analyzer (Japan). GFR was measured with use of CKD-EPI formula (2021 Creatinine) [53]. Chromogranin A (CgA) was measured using the LDN Company ELISA test (Germany). The sensitivity of the method for this parameter was 1.4 µg/L. Morphology was evaluated using the Sysmex Corporation XN 1000 automatic hematology analyzer (Japan). The reference ranges for the laboratory tests discussed in the paper are presented below in Table 8.

### 4.2. Statistical Analysis

Statistical analysis was performed using Statistica 12 software (StatSoft, Inc., Cravov, Poland, 2021). To verify whether the results met the rules of normal distribution, the Shapiro–Wilk test was conducted. Results with normal distribution were presented as means (M) and standard deviations (SD), and in the case of non-normal distribution as medians (Med.) and interquartile ranges (IQR). Differences between groups were analyzed using appropriate tests, such as the t-Student tests, Wilcoxon test, and Mann–Whitney test. A significance level of *p* < 0.05 was adopted.

## 5. Conclusions

Our study showed that, immediately after RLT (87.5%) and in long-term observation, 51% of NEN patients of unknown origin, located outside the midgut and pancreas, and ones with G3, benefited (by partial regression or disease stabilization) from the radioligand therapy with 177-Lutetium alone or tandem therapy with 177-Lutetium and 90-Yttrium. The therapy demonstrated no (significant) adverse effects on kidney function, while deterioration of bone marrow parameters was within acceptable range. Decrease of chromogranin concentration proved to be a good indicator of disease regression or stabilization. Therefore, we believed that radioligand therapy should be considered as a treatment option for all NEN patients with preserved somatostatin receptors expression.

## Figures and Tables

**Table 1:** Physical features of radioisotopes.

	^177^Lu	^90^Y
Emax [MeV]	0.497	2.27
Range of action [mm]	2	11
Half-life [days]	6.647	2.67

Emax—maximum energy, MeV—millielectronvolts, mm—millimeters, ^177^Lu—177-Lutetium, ^90^Y—90-Yttrium.

**Table 2:** Treatment timelines analysis.

Age at NEN Diagnosis [years]	MEAN	55.31
	SD	12.69
Age at RLT start [years]	MEAN	58.53
	SD	12.44
Time from NEN diagnosis to RLT [months]	MEAN	34.39
	SD	35.88

**Table 3:** Comparison of median and mean progression free survival of the study group during somatostatin analogues treatment—before RLT qualification.

	Median	IQR	Mean	SD
PFS_Un_ (*n* = 25)	19	23	26.19	21.00
PFS_Oth_ (*n* = 21)	31	28	40.55	42.35
PFS_G3_ (*n* = 7)	18	40	38.00	41.80

PFS_Un_—progression free survival in subgroup of unknown primary tumor location, PFS_Oth_—progression free survival in subgroup of other than GEP tumor location, PFS_G3_—progression free survival in subgroup of G3 NEN.

**Table 4:** The data of “progression” subgroup.

Patient	Gender	Age	Staging	Location	Function	Ki-67	Previous Surgery	Previous Chemo	SSA	Treatment
1	M	49	G3	Lung	carcinoid	25	No	Yes	Lanreotide	Lu
2	M	32	G1	Lung	carcinoid	2	Yes	Yes	Lanreotide	Lu
3	M	63	G1	Unknown	carcinoid	2	No	Yes	Lanreotide	Lu/Y
4	M	50	G2	Unknown	carcinoid	15	No	No	Octreotide	Lu/Y
5	F	62	G2	Unknown	non-functioning	6	Yes	No	Lanreotide	Lu
6	F	60	G3	Pancreas	non-functioning	25	No	No	Lanreotide	Lu

**Table 5:** Long-term observation data of the group. OS—overall survival, PFS—progression free survival, OT—observation time, Ki-67—proliferation index, NA—not applicable.

	Number of Patients	Median OT	IQR
Whole available group (OT)	43	14	18
Stabilization subgroup (OT)	26	15	17
Progression (PFS)Death (OS)	8	20	16
Death (OS)	9	8	14
Status unknown	8	NA	NA

**Table 6:** Means and medians of laboratory parameters in the subgroup treated with 177-Lutetium and 177-Lutetium and 90-Yttrium before first course (A) versus before last course (B) of RLT.

Parameters	^177^Lu A (*n* = 32)		^177^Lu B (*n* = 32)		*p*	^177^Lu and ^90^Y A (*n* = 16)		^177^Lu and ^90^Y B (*n* = 16)		*p*
	Mean	SD	Mean	SD		Mean	SD	Mean	SD	
GFR	80.25	20.47	79.03	21.78	0.268	86.38	16.99	86.81	19.39	0.419
CREA	0.97	0.23	0.98	0.27	0.272	0.85	0.15	0.86	0.16	0.396
RBC	4.25	0.48	3.86	0.47	<0.001	4.39	0.67	3.88	0.63	<0.001
WBC	7.07	3.11	5.56	2.42	0.002	6.49	2.61	4.17	1.7	<0.001
PLT	280.72	118.08	229.75	99.77	0.022	218.38	63.84	176.75	58.72	0.012
HGB	12.57	1.31	11.96	1.14	<0.001	12.97	1.33	12.1	1.38	0.002
Parameters	Median	IQR	Median	IQR	*p*	Median	IQR	Median	IQR	*p*
CgA	258	2238	126	1103	0.004	329	865	166	640	0.004

GFR—glomerular filtration rate, CREA—creatinine, RBC—red blood cells, WBC—white blood cells, PLT—platelets, HGB—hemoglobin, CgA—chromogranin A, *p*—*p*-value, ^177^Lu—177-Lutetium; ^90^Y—90-Yttrium.

**Table 7:** Comparison of change (Δ) of laboratory parameters in patients treated with ^177^Lu versus ^177^Lu and^90^Y.

	^177^Lu		^177^Lu and ^90^Y		*p*
Δ	Mean	SD	Mean	SD	
GFR	−1.22	10.84	0.44	8.12	0.599
CREA	0.02	0.14	0.01	0.09	0.814
RBC	−0.39	0.32	−0.51	0.41	0.296
WBC	−1.51	2.65	−2.32	2.08	0.301
PLT	−50.97	134.55	−41.63	64.59	0.797
HGB	−0.61	0.83	−0.87	0.98	0.359
Δ	Median	IQR	Median	IQR	*p*
CgA	−31	1025	−49	296	0.561

GFR—glomerular filtration rate, CREA—creatinine, RBC—red blood cells, WBC—white blood cells, PLT—platelets, HGB—hemoglobin, CgA—chromogranin A, *p*—*p*-value, Δ—change. ^177^Lu—177-Lutetium; ^90^Y—90-Yttrium/.

**Table 8:** Reference ranges of blood parameters.

Parameters	Reference Range
WBC [1000/µL]	4.0–10.0
RBC [mln/µL]	3.5–5.5
HGB [g/dL]	11.0–18.0
PLT [1000/µL]	150–400
CREA [mg/dL]	0.7–1.2
GFR [mL/min/1.73 m^2^]	>90
CgA [ng/mL]	19–100

WBC—leucocytes, RBC—erythrocytes, HGB—hemoglobin, PLT—blood platelets, CREA—serum creatynine CgA—chromogranin A.

## Data Availability

The datasets used and/or analyzed during the current study are available from the corresponding author on reasonable request.
